# Minimally-invasive Distal Metatarsal Diaphyseal Osteotomy in the Treatment of Plantar Ulcer in the Diabetic Foot: A Case Report

**DOI:** 10.1055/s-0044-1790194

**Published:** 2024-12-27

**Authors:** Fernando Delmonte Moreira, Jorge Eduardo de Schoucair Jambeiro, Antero Tavares Cordeiro Neto, Roger Carneiro Dourado, Eduardo Carrilho Padula, Alex Guedes

**Affiliations:** 1 Grupo de Cirurgia do Pé e Tornozelo, Hospital Santa Izabel, Santa Casa de Misericórdia da Bahia, Salvador, BA, Brasil; 2 Serviço de Pé e Tornozelo, Hospital Universitário Pedro Ernesto, Universidade do Estado do Rio de Janeiro, Rio de Janeiro, RJ, Brasil; 3 Grupo de Oncologia Ortopédica, Hospital Santa Izabel, Santa Casa de Misericórdia da Bahia, Salvador, BA, Brasil

**Keywords:** diabetes mellitus, diabetic foot, foot deformities, foot ulcer, osteotomy, surgical procedures, operative

## Abstract

The diabetic foot consumes a large number of resources and has a profound negative impact on quality of life, representing the major non-traumatic cause of lower limb amputation in adults. The present report describes a diabetic patient with a recurrent plantar ulcer in the topography of the heads of the second, third, and fourth metatarsals. The patient was treated using the distal metatarsal diaphyseal osteotomy (DMDO) technique in these bones, an Akin-type percutaneous osteotomy in the proximal phalanx of the hallux, and debridement. The 5-year postoperative follow-up revealed good outcomes regarding healing and prevention of new episodes.

## Introduction

Approximately 25% of hospital admissions of diabetic patients directly result from foot problems. 1 Foot ulcers occur in approximately 15% of diabetic patients with peripheral neuropathy, leading to numerous complications, including abscesses and deep infection (osteomyelitis).

The risk of death for diabetic patients with foot ulcers is 2.5 times higher compared with that of diabetic subjects without this complication, with a 5% mortality rate during the 1 ^st^ year and a 42% mortality rate within 5 years of its onset. 2

About 71 to 85% of patients with recurrent plantar ulcers require mutilating procedures; 3 the risk is 15 to 40 times higher than in non-diabetics, 1 making diabetes the major cause of non-traumatic lower limb amputations. However, amputations may not be the most effective solution to this condition, as they may entail potential postoperative complications, including infections, which, in diabetic patients, tend to be polymicrobial.

Percutaneous/minimally-invasive surgery (MIS) represents one of the most innovative approaches to the foot and ankle. 3 As MIS is less traumatic and does not require osteosynthesis, it reduces the risk of infections, vascular complications, and healing problems, especially in diabetic patients. 4 However, since it does not enable the direct visualization of anatomical structures, it is critical to become familiar with the technique and its instruments (drills, motor, scalpel blades, and detachers) under fluoroscopy control and to have in-depth knowledge of the local anatomy to avoid iatrogenic injuries. 5

The current case report presents a diabetic patient with a chronic, infected plantar ulcer in the topography of the heads of the second, third, and fourth metatarsals of the right forefoot treated using the distal metatarsal diaphyseal osteotomy (DMDO) technique 2 in these bones, an Akin-type 5 percutaneous osteotomy in the proximal phalanx of the hallux, and debridement.

## Case Report

The institutional Ethics Committee approved the present case report under CAAE number 44105521.6.0000.5520. The patient signed the required informed consent form.

**Fig. 1:**
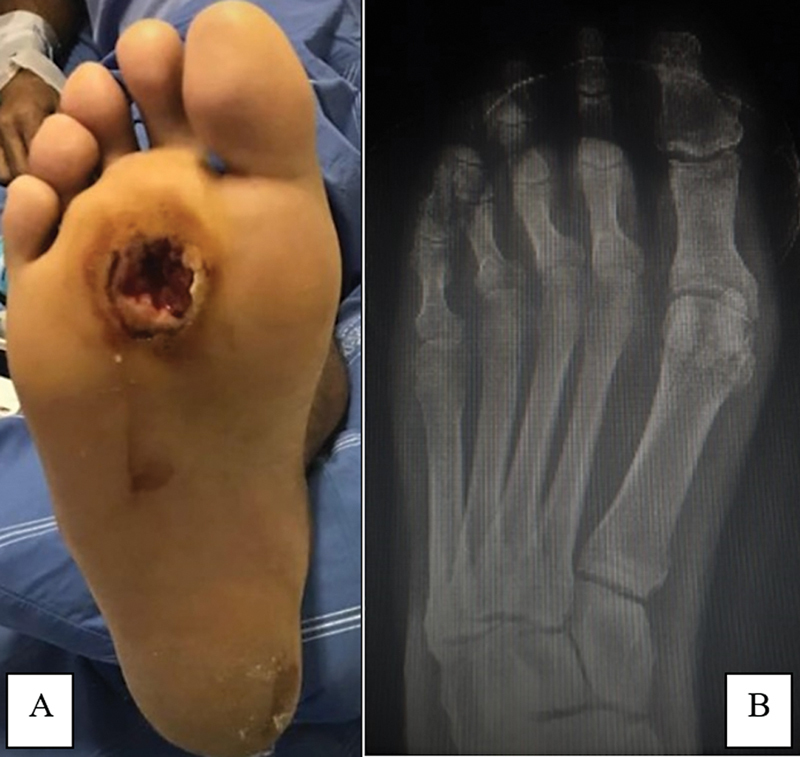
Clinical and radiographic appearance of the right foot on the first visit. ( **A** ) Ulcer on the topography of the central metatarsal heads; ( **B** ) anteroposterior (AP) radiograph of the right foot showing no signs of bone involvement/osteomyelitis.

The patient was male, white, aged 58 years. He was a rural worker, and the diabetes diagnosis had occurred 16 years before. The patient underwent clinical monitoring with irregular use of insulin and metformin. Upon admission, he had increased blood glucose levels (280 mg/dL), chronic renal failure, systemic arterial hypertension, and pulmonary sarcoidosis. However, he did not present significant vascular changes. He presented to our service with a recurrent neuropathic infected plantar ulcer in the topography of the heads of the 2 ^nd^ , 3 ^rd^ , and 4 ^th^ metatarsals of the right foot, with approximately 4 cm in diameter ( Fig. 1 ). The patient also had an overloaded area and valgus deformity of the hallux.

The radiographs requested during our care did not show signs of osteomyelitis ( Fig. 1 ), which is consistent with the magnetic resonance imaging (MRI) scans brought by the patient from another service.

The ulcer was of grade 2b per the University of Texas classification ( Table 1 ). Given the condition, we recommended urgent surgery, but the patient refused hospitalization. Three days later, with his condition worsening, he returned to the unit and underwent admission. The surgery occurred under spinal anesthesia, with the performance of the DMDO technique on the second, third, and fourth metatarsals, an Akin-type percutaneous osteotomy, and ulcer debridement. We collected ulcer samples for culture with antibiogram. At the end of the procedure, we placed simple bandages over the wounds and immobilized the fingers with tape.

**Table 1:** University of Texas classification of diabetic ulcers

Stage	Grade			
	0	I	II	III
**A (absence of infection or ischemia)**	Completely epithelialized pre- or postulcerative lesion	Superficial wound not involving the tendon, capsule, or bone	Wound with tendon or capsule exposure	Wound with bone or joint exposure
**B**	Infection	Infection	Infection	Infection
**C**	Ischemia	Ischemia	Ischemia	Ischemia
**D**	Infection and ischemia	Infection and ischemia	Infection and ischemia	Infection and ischemia

After the procedure, the patient received empirical antibiotic therapy (cefazolin, 1 g every 8 hours) intravenously (IV) for 3 days until the culture results, which were positive for *Pseudomonas aeruginosa* (sensitive to ciprofloxacin). Therefore, we changed the antibiotic to IV ciprofloxacin (400 mg every 12 hours) and extended the hospital stay for another 7 days. Next, we discharged the patient with a prescription for oral antibiotic therapy (ciprofloxacin, 500 mg every 12 hours) for another 3 months, indicating outpatient follow-up with the endocrinology and infectious disease teams.

We allowed immediate walking without load restrictions on the operated limb and instructed the patient to use rigid-soled orthopedic sandals for 6 weeks. Dressings and immobilization changes occurred every other day during the first week. After that, we changed the immobilization weekly until the sixth postoperative week.

**Fig. 2:**
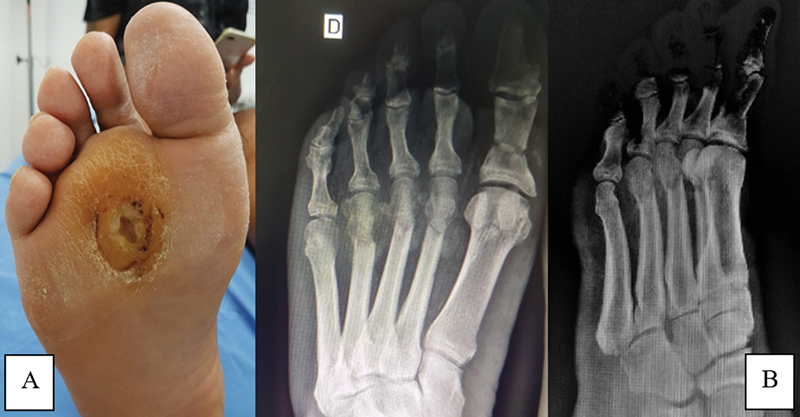
Clinical and radiographic appearance of the right foot in the third postoperative week. ( **A** ) Progression of the plantar ulcer aspect; ( **B** ) AP and oblique radiographs of the right foot demonstrating signs of consolidation of the osteotomy foci.

**Fig. 3:**
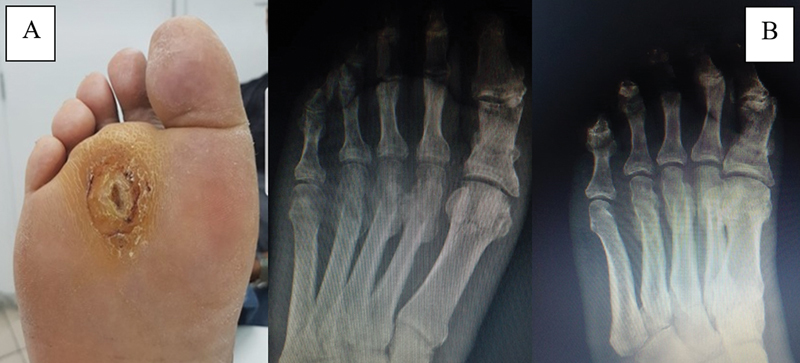
Clinical and radiographic appearance of the right foot in the third month after surgery. ( **A** ) Progression of the plantar ulcer aspect; ( **B** ) AP and oblique radiographs of the right foot.

At the first dressing changes, we observed an improved wound appearance. This improvement progressed after discharge, with good ulcer healing and osteotomy consolidation ( Fig. 2 ). In the third postoperative month, we noted complete ulcer healing and full consolidation of the osteotomy foci ( Fig. 3 ).

**Fig. 4:**
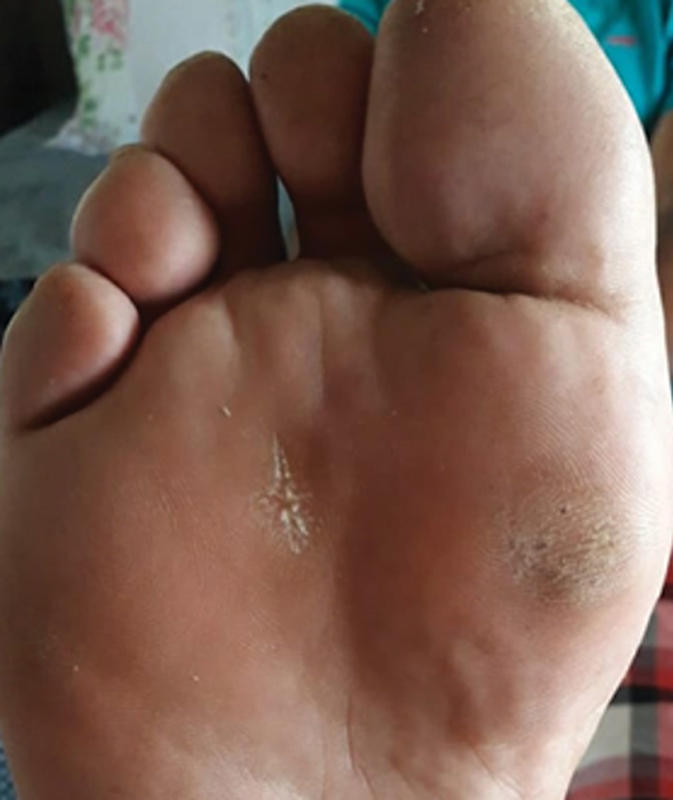
Progression of the plantar ulcer aspect in the 5 ^th^ postoperative year.

Currently, the patient is in the fifth postoperative year, with no ulcer recurrence or infection signs in the forefoot and no complaints of local pain or transfer metatarsalgia ( Fig. 4 ).

## Discussion

Diabetes mellitus is a chronic disease with a high rate of morbidity and mortality. 1 The literature 6 presents disappointing functional outcomes in the management of diabetic neuropathic ulcers. Furthermore, the presence of a plantar ulcer precedes 85% of cases of lower-limb amputation in patients with diabetes. 7

The treatment of diabetic foot ulcers remains a challenge. It includes preventive clinical measures (adequate glycemic control, optimization of the nutritional status, total smoking cessation, and improvement in limb circulation), debridement, removal of the support load on the foot (using sandals with wedged soles, removable boots, walkers, custom orthotics, and full contact cast), and frequent dressings. Hyperbaric oxygen therapy and negative pressure therapy have been advocated as advanced modalities to accelerate wound healing. 8

Inclusion of the MIS in the therapeutic arsenal improved the management of these injuries, leading to outcomes that were highly uncertain until a few years ago. 2 The DMDO technique relies on osteotomy proximal to the metatarsal neck. It is indicated to reduce the pressure over the ulcer to favor its healing and enable an improvement in biomechanics by restoring a balanced harmonic forefoot arch. This technique protects diabetic patients with minimal tissue damage, enabling immediate postoperative load and reducing the risk of infections, since it does not require implants. 2 Nevertheless, this procedure is contraindicated in subjects with cellulitis or ischemic foot.

Although there are few reports on the use of minimally invasive techniques in the treatment of plantar ulcers in diabetics, the outcomes have been promising, offering a solution and eventually interrupting the progression to more severe stages. 2 3 4 9 10 When they occur, complications include recurrence, transfer ulcer, 10 superficial infections, malunion, and pseudarthrosis. However, the rates of these complications are lower compared with those of the conventional techniques. 2

The procedure proved to be effective, without complications, and there was no recurrence of the plantar ulcer during the follow-up period, which is consistent with the relevant literature.
