# Supplementary material for: Minimally-invasive Distal Metatarsal Diaphyseal Osteotomy in the Treatment of Plantar Ulcer in the Diabetic Foot: A Case Report
Source: Rev Bras Ortop (Sao Paulo). 2024 Dec 27;59(Suppl 2):e228–32. doi: 10.1055/s-0044-1790194 (PMC11679632; doi:10.1055/s-0044-1790194)
Supplement: Related texts

# Research article: Osteotomia metatarsal diafisária distal minimamente invasiva no tratamento de úlcera plantar no pé diabético: Um relato de caso

**Keywords:** deformidades do pé, diabetes mellitus, osteotomia, pé diabético, procedimentos cirúrgicos operatórios, úlcera do pé

## Abstract

O pé diabético consome grande quantidade de recursos e tem profundo impacto negativo sobre a qualidade de vida, pois representa a principal causa não traumática de amputação dos membros inferiores em adultos. Este relato apresenta um paciente diabético, portador de úlcera plantar recidivante na topografia das cabeças do segundo, terceiro e quarto metatarsos. O paciente foi tratado mediante a técnica de osteotomia metatarsal diafisária distal ( *distal metatarsal diaphyseal osteotomy* , DMDO, em inglês) nesses ossos, associada à osteotomia percutânea tipo Akin na falange proximal do hálux e desbridamento. No seguimento pós-operatório de 5 anos, o paciente apresentou resultados bons no que diz respeito à cicatrização e à prevenção de novos episódios.

## Introdução

Aproximadamente 25% das internações hospitalares em diabéticos relacionam-se diretamente com problemas nos pés. 1 As úlceras podálicas ocorrem em aproximadamente 15% dos diabéticos que apresentam neuropatia periférica, o que leva a inúmeras complicações, como abcessos e infecção profunda (osteomielite).

Pacientes diabéticos com úlceras podálicas apresentam risco de óbito 2,5 vezes maior do que diabéticos sem essa complicação, com 5% de mortalidade durante o 1° ano e 42% de mortalidade em 5 anos após o seu surgimento. 2

Procedimentos mutilantes são necessários em 71 a 85% dos pacientes portadores de úlceras plantares recorrentes; 3 o risco é de 15 a 40 vezes maior do que em não diabéticos, 1 o que torna o diabetes a principal causa de amputações não traumáticas dos membros inferiores. No entanto, as amputações podem não constituir a solução mais eficaz diante desta condição, pois apresentam potencial para complicações pós-operatórias, como infecções, que, em diabéticos, tendem a ser polimicrobianas.

A cirurgia percutânea/minimamente invasiva ( *minimally-invasive surgery* , MIS, em inglês) representa uma das abordagens mais inovadoras para o pé e o tornozelo. 3 Por ser menos traumática e não demandar osteossíntese, diminui o risco de infecções, complicações vasculares e problemas de cicatrização, sobretudo em diabéticos. 4 Entretanto, como as estruturas anatômicas não são visualizadas diretamente, é fundamental familiarizar-se com a técnica e a utilização do instrumental (brocas, motor, lâminas de bisturi e descoladores) sob controle de fluoroscopia e ter conhecimento profundo da anatomia local, para evitar lesões iatrogênicas. 5

Este relato de caso apresenta paciente diabético, portador de úlcera plantar crônica infectada na topografia das cabeças do segundo, terceiro e quarto metatarsos do antepé direito, tratada mediante técnica de osteotomia metatarsal diafisária distal ( *distal metatarsal diaphyseal osteotomy* , DMDO, em inglês) 2 nesses ossos, associada à osteotomia percutânea tipo Akin 5 na falange proximal do hálux e desbridamento.

## Relato do Caso

O relato de caso foi aprovado pelo Comitê de Ética da nossa instituição sob o número CAAE: 44105521.6.0000.5520, e o termo de consentimento livre e esclarecido foi assinado pelo paciente.

Trata-se de paciente do sexo masculino, branco, de 58 anos, trabalhador rural, diabético com diagnóstico havia 16 anos, em acompanhamento clínico e uso irregular de insulina e metformina. À admissão, ele apresentava glicemia aumentada (280 mg/dL), além de insuficiência renal crônica, hipertensão arterial sistêmica e sarcoidose pulmonar; no entanto, não apresentava alterações vasculares significativas. O paciente procurou nosso serviço apresentando úlcera plantar neuropática de repetição infectada na topografia das cabeças do 2°, 3° e 4° metatarsos do pé direito, com aproximadamente 4 cm de diâmetro ( Fig. 1 ). Ele também apresentava área de sobrecarga e deformidade em valgo do hálux.

**Fig. 1:** Aspectos clínico e radiográfico do pé direito no primeiro atendimento. ( **A** ) Úlcera na topografia das cabeças dos metatarsos centrais; ( **B** ) radiografia em incidência anteroposterior (AP) do pé direito, sem indícios de comprometimento ósseo/osteomielite.

As radiografias realizadas durante o nosso atendimento não apresentavam indícios de osteomielite ( Fig. 1 ), assim como o exame de ressonância magnética (RM) trazido de outro serviço pelo paciente.

A úlcera foi classificada como de grau 2b da University of Texas ( Tabela 1 ). Diante do quadro, foi indicada cirurgia de urgência, mas o paciente recusou a internação. Três dias depois, com a piora do quadro, ele retornou à unidade, sendo então hospitalizado. Conforme já mencionado, o paciente foi operado sob raquianestesia mediante a técnica de DMDO, 2 realizada no segundo, terceiro e quarto metatarsos, associada à osteotomia percutânea tipo Akin e ao desbridamento da úlcera. Foram colhidas amostras para cultura com antibiograma. Ao final do procedimento, foram feitos curativo simples dos ferimentos e imobilização dos dedos com esparadrapos.

**Tabela 1:** Classificação da University of Texas para úlceras diabéticas

Estágio	Grau			
	0	I	II	III
**A (ausência de infecção ou isquemia)**	Lesão pré ou pós-ulcerativa completamente epitelizada	Ferida superficial que não envolve tendão, cápsula ou osso	Ferida com exposição de tendão ou cápsula	Ferida com exposição de osso ou articulação
**B**	Infecção	Infecção	Infecção	Infecção
**C**	Isquemia	Isquemia	Isquemia	Isquemia
**D**	Infecção e isquemia	Infecção e isquemia	Infecção e isquemia	Infecção e isquemia

Após o procedimento, o paciente fez uso de antibioticoterapia empírica (cefazolina 1 g de 8/8 h) intravenosa (IV) por 3 dias, até que saíram os resultados das culturas, positivas para *Pseudomonas aeruginosa* (sensível ao ciprofloxacino). Diante disso, foi feita a troca do antibiótico para ciprofloxacino IV (400 mg de 12/12 h), e a permanência hospitalar ampliada para mais 7 dias. Após esse período, o paciente teve alta, a antibioticoterapia foi mantida por via oral (ciprofloxacino 500 mg de 12/12 h) por mais 3 meses, e indicou-se acompanhamento ambulatorial com as equipes de endocrinologia e infectologia.

Foi permitida a deambulação imediata, sem restrição de carga no membro operado, com o uso de sandália ortopédica de solado rígido por 6 semanas. Os curativos e a imobilização foram trocados em dias alternados na primeira semana; após isso, a imobilização foi trocada semanalmente até a sexta semana pós-operatória.

Já nas primeiras trocas de curativos, observou-se melhora do aspecto da ferida, que progrediu após a alta, com boa evolução no que diz respeito à cicatrização da úlcera e à consolidação das osteotomias ( Fig. 2 ). No terceiro mês de pós-operatório, a úlcera já apresentava cicatrização completa, e os focos de osteotomia encontravam-se totalmente consolidados ( Fig. 3 ).

**Fig. 2:** Aspectos clínico e radiográfico do pé direito na terceira semana de pós-operatório. ( **A** ) Aspecto evolutivo da úlcera plantar; ( **B** ) radiografias nas incidências AP e oblíqua do pé direito, que demonstram indícios de consolidação dos focos de osteotomia.

**Fig. 3:** Aspectos clínico e radiográfico do pé direito no terceiro mês de pós-operatório. ( **A** ) Aspecto evolutivo da úlcera plantar; ( **B** ) radiografias nas incidências AP e oblíqua do pé direito.

Atualmente, o paciente encontra-se no quinto ano de pós-operatório, sem recidiva da úlcera ou sinais de infecção no antepé, e sem queixas álgicas locais ou metatarsalgia de transferência ( Fig. 4 ).

**Fig. 4:** Aspecto evolutivo da úlcera plantar no 5° ano de pós-operatório.

## Discussão

O diabetes mellitus constitui doença crônica associada a alta taxa de morbidade e mortalidade. 1 A literatura 6 apresenta resultados funcionais decepcionantes no manejo das úlceras neuropáticas diabéticas. Além disso, a presença de úlcera plantar precede 85% dos casos de amputação do membro inferior nos portadores dessa doença. 7

O tratamento das úlceras do pé diabético ainda hoje constitui desafio, pois inclui, além de medidas clínicas preventivas (controle glicêmico adequado, otimização do estado nutricional, interrupção total do tabagismo, e melhora na circulação da extremidade), o desbridamento, a retirada da carga de apoio sobre o pé (uso de sandálias com solado elevado em cunha, botas removíveis, andadores, órteses feitas sob medida, gesso de contato total) e curativos frequentes. A oxigenoterapia hiperbárica e a terapia que utiliza pressão negativa vêm sendo defendidas como modalidades avançadas para acelerar a cicatrização das feridas. 8

A inclusão da MIS no arsenal terapêutico melhorou o manejo dessas lesões, o que levou a resultados que, até poucos anos atrás, seriam altamente incertos. 2 A técnica de DMDO baseia-se na osteotomia proximal ao colo metatarsal, indicada para a redução da pressão sobre a úlcera, o que favorece a sua cicatrização, além de possibilitar a melhora da biomecânica por meio da restauração de um arco harmônico equilibrado do antepé. Essa técnica protege os pacientes diabéticos com mínimo dano tecidual, possibilita carga pós-operatória imediata, e apresenta risco reduzido de infecções, pois não requer a utilização de implantes; 2 entretanto, este procedimento está contraindicado na presença de celulite ou pé isquêmico.

Apesar de haver poucos relatos do uso de técnicas minimamente invasivas no tratamento das úlceras plantares em diabéticos, os resultados demonstrados têm sido promissores, pois oferecem solução e acabam por interromper a evolução para estágios mais graves. 2 3 4 9 10 Quando ocorrem, as complicações incluem recidiva, úlcera de transferência, 10 infecção superficial, consolidação viciosa e pseudartrose; entretanto, elas ocorrem em menor percentual quando comparadas à utilização de técnicas convencionais. 2

O procedimento realizado se mostrou eficaz, sem complicações, e não houve recidiva da úlcera plantar no período de acompanhamento, o que está de acordo com a literatura pertinente.
